# Dietary fatty acid source has little effect on the development of the immune system in the pyloric caeca of Atlantic salmon fry

**DOI:** 10.1038/s41598-018-37266-3

**Published:** 2019-01-10

**Authors:** Mahsa Jalili, Yang Jin, Atle M. Bones, Yngvar Olsen, Olav Vadstein, Mari-Ann Østensen, Francesco Buonocore, Marco Gerdol, Alberto Pallavicini, Giuseppe Scapigliati

**Affiliations:** 1 Department of Biology, NTNU Norwegian University of Science and Technology, N-7491 Trondheim, Norway; 2 Department of Biotechnology and Food Science, NTNU Norwegian University of Science and Technology, N-7491 Trondheim, Norway; 3 Department for Innovation in Biological, Agro-food and Forest systems (DIBAF), University of Tuscia, 01100 Viterbo, Italy; 4 Department of Life Sciences, University of Trieste, 34100 Trieste, Italy; 5 Present Address: Faculty of Biosciences, Department of Animal and Aquacultural Sciences, Centre for Integrative Genetics, Norwegian University of Life Sciences, Ås, Norway

## Abstract

The quality and relative amounts of dietary lipids may affect the health and growth of cultured Atlantic salmon. So far, little is known about their effects on the performance of the fish immune system during early life stages and, in particular their importance in the transition from endogenous nutrition (yolk) in the alevin stage to exogenous nutrition in the later fry stage. We investigated the immunomodulatory effects of fish oil, vegetable oil and phospholipid-rich oil in feeds for farmed Atlantic salmon using a transcriptomic approach. The experiment allowed a fine-scale monitoring of gene expression profiles in two tissues, the pyloric caeca of the intestine and the liver, in a 94 days-long first feeding experiment. The analysis of transcriptional profiles revealed that first feeding induced a strong immunomodulation in the pyloric caeca after 48 days of feeding, lasting up to day 94 and possibly beyond. On the other hand, the differential effect of the three dietary regimes was negligible. We interpret this upregulation, undetectable in liver, as a potentiation of the immune system upon the first contact of the digestive system with exogenous feed. This process involved a complex network of gene products involved in both cellular and humoral immunity. We identified the classical pathway of the complement system, acting at the crossroads between innate and adaptive immunity, as a key process modulated in response to the switch from endogenous to exogenous nutrition.

## Introduction

The feeding regime in fish farming is a crucial factor in ensuring the health of animals and improving the quality of product for human nutrition. The aquaculture production of Atlantic salmon (*Salmo salar*) currently exceeds 2,000,000 tons per year, accounts for over 50% of the global production of salmonids and is rapidly growing worldwide^1^. The requirements of this important sector include a continuous and adequate feed supply, to achieve a high quality, safe and sustainable animal product. Indeed, fish nutrition and the connected issues of overfeeding and waste production may have a direct impact both on fish and consumer welfare, and on the ecological quality of the environment^2^.

Although fish oil has been traditionally used as the main source of long chain polyunsaturated fatty acids (LC-PUFAs) in the aquaculture industry, the use of vegetable oil as a substitute increased significantly in recent years^3^. Vegetable oil is devoid of LC-PUFA, but often contains high amounts of linoleic acid (LNA) or alpha-linolenic acid (ALA)-rich, which serve as sources of n-6 and n-3 fatty acids, respectively^4^. The amount of LC-PUFAs present in fish tissues is related to the intake of dietary fatty acids^4^. The content of phospholipids in the feeds is another aspect which needs to be taken into account, as they are key components of cell membranes and indispensable for normal growth and development, homeostasis^5^ and modulation of immune defense against pathogens^6^.

Over the years, several studies have pointed out that the quality of diet, in terms of phospholipid and fatty acid (FA) contents, may have important effects on the immune response of fish. This can be conveniently tracked by investigating the modulation of expression profiles of immune genes^7–9^. However, the biological interpretation of gene expression data in the context of feeding is not straightforward, as the intensity and significance of modulation depends on various factors. These include dietary ingredients, the duration of the experimental study, as well as the species, developmental stages and tissues studied^10–13^ and, crucially, the effects on gut microbiota^14^. Despite these uncertainties, several studies are concordant in supporting the use of feed supplements as nutraceuticals in fish aquaculture, due to their ability to modulate the immune system since the early life stages^15^,

The Atlantic salmon has been the subject of extensive studies on the effects of nutrients intake (e.g. lipids, carbohydrates, vitamin B6, C and A) and frequency of feeding on the innate immune response^7,9,16^. In particular, the transcriptional effects of dietary supplementation with plant-based ingredients such as glucosinolates, n-3 FAs, vitamins and phosphorous compounds, have been investigated in parasite-infested salmons. The results supported an up-regulation of immune-related genes, always coupled with a general amelioration of the infection status, although the specific molecules involved varied depending on the tissues studied^17–19^. On the other hand, it is well established that some dietary supplements have nearly no significant effect on transcription in the digestive tract^20^ ad that others, such as solvent-extracted soybean meal, may even have a negative impact on fish health due to the development of enteropathy^21^. Remarkably, intestinal inflammation can be reverted by the use of microbial feed additives^22^.

Only a handful of studies have so far studied the modulation of the salmon immune system during the early stages of development in response to different diets. The most significant contribution in this context is represented by the work of Sahlmann and colleagues^23^. They monitored the morpho-histological developments of different organs and the expression levels of several genes by qPCR during the first 144 days post-hatching, comparing a soybean meal diet and a control fishmeal-based diet. Besides providing a detailed timeline for the morphological development of the digestive system, that study also revealed that the soybean meal diet could not induce inflammation in the intestinal tract of juvenile salmons. Despite the growing body of knowledge concerning the relationship between diet and immune response in farmed salmon, a comparative assessment of the modulation of the immune system in response to different feeding regimes is still lacking.

Important efforts in the genetic and molecular studies of the Atlantic salmon have led to the release of a fully annotated reference genome^24^, a resource of the utmost importance for basic biological studies on rediploidization, as well as a reference for large scale genotyping^25^ and epigenetics studies^26^. Most importantly, it can serve as a reference for transcriptome profiling, with a broad range of applications^27–31^.

We applied a whole-transcriptome sequencing approach to *S. salar*, with the aim to pinpoint the most significant changes in the expression of immune-related genes, in response to: a) phospholipid-rich lipids of animal origin (PL); b) lipids of plant origin (VO); c) fish oil-based feeds (FO). The experiment focus on the transition between the alevin and fry developmental stages, concurrent with the internalization of the yolk sac and the switch from endogenous to exogenous nutrition, and allowed the monitoring of gene expression profiles over a period of 94 days.

## Material and Methods

### Sampling of animals and RNA-sequencing

Fertilized Atlantic salmon (*Salmo salar*) eggs (Aquaculture strain) were shipped from the AquaGen salmon breeding company to the Ervik hatchery (Frøya, Norway). The experiment was started by nourishing salmon fry (47 days post hatching), immediately after the internalization of the yolk sac, by start feeding on three different pellet diets supplemented with: fish oil (FO), phospholipid-rich animal and vegetable-based oil (PL) and vegetable oil (VO). The ingredients are shown in Table 1. The source of phospholipids was krill and herring roe (50/50). The percentage of docosahexaenoic acid (DHA) of total fatty acids in the diets were 17%, 3.7%, 11% in FO, VO and PL diets respectively. The feeds were produced by Sparos (Olhão, Portugal). The fish were reared in six indoor freshwater tanks (3 groups × 2 replicate tanks), with 200 individuals each, from first feeding and for 94 days. Four salmon fry individuals per group (n = 2 × 2) were sampled at day 0 (mean weight 0.23 g, before the start of the feeding experiment), day 48 (~1.3 g) and day 94 (~4.5 g) after first feeding. The fish were sacrificed by exposure to 200 mg/ml Benzoak vet. (ACD Pharmaceuticals AS, Oslo, Norway), following manufacturer’s instructions. The pyloric caeca (PC) and liver (LV) tissues were dissected in a sterile petri dish under a dissecting microscope, immediately stored in RNA later solution, placed for 24 h at 4 °C to allow sufficient penetration of the solution into the tissues, and then kept at −80 °C until RNA extraction.

**Table 1:** Feed composition of the three *Salmo salar* diets employed in the present study.

Feedings ingredients	FO	VO	PL
Percentage for a whole meal	%	%	%
Fishmeal 70 LT FF Skagen	10.00	10.00	10.00
Fish protein concentrate (CPSP 90)	15.00	15.00	15.00
Squid meal	25.00	25.00	25.00
Shrimp hydrolysate	5.00	5.00	5.00
Fish gelatin	2.00	2.00	2.00
Pea protein concentrate	7.50	7.50	7.50
Wheat Gluten	12.50	12.50	12.50
Potato starch gelatinized	2.50	2.50	2.50
Fish oil	7.20	0.00	3.00
Tuna oil	2.30	0.00	0.00
Rapeseed oil	0.00	2.90	2.50
Linseed oil	0.00	2.40	0.00
Palm oil	0.00	4.20	0.00
Vitamin & Mineral Premix	1.50	1.50	1.50
Lutavit C35	0.03	0.03	0.03
Lutavit E50	0.12	0.12	0.12
Brewer’s yeast	5.00	5.00	5.00
Betaine HCl	1.00	1.00	1.00
MAP (mono-ammonium phosphate)	3.00	3.00	3.00
L-Taurine	0.35	0.35	0.35
NTNU - Phospholipids	0.00	0.00	4.00
Total	100.00	100.00	100.00

The RNA extraction was performed with the RNeasy Plus Universal Mini Kit (Qiagen, Hilden, Germany), according to the manufacturer’s protocol. The concentration and integrity of RNA were determined by a Nanodrop 8000 (Thermo Fisher Scientific, Waltham, USA) and a 2100 Bioanalyzer (Agilent Technologies, Santa Clara, USA), respectively. All RNA samples had RNA integrity (RIN) values higher than 8, which is sufficient for transcriptome analysis. Sequencing libraries were prepared with a TruSeq Stranded mRNA Library Prep Kit (Illumina, San Diego, USA) according to the manufacturer’s protocol. Libraries were sequenced on a HiSeq. 2500 platform (Illumina, San Diego, USA) at the Norwegian Sequencing Facility (Oslo, Norway), using a single-end 120 cycles strategy.

### Gene expression analysis

The entire pipeline of RNA-seq data analysis was performed with the CLC Genomics Workbench, version 10 (Qiagen, Hilden, Germany). The raw sequencing output, deposited in the NCBI SRA database, was processed to remove low quality bases (with threshold 0.05), residual Illumina Truseq adaptors and the reads shorter than 90 bp. Each individual set of reads was mapped against the annotated Salmon reference genome (http://salmobase.org/download.html) with the RNA sequencing mapping algorithm, using the following parameters: length fraction 0.75, similarity fraction 0.98. Unique gene reads counts were used for subsequent analyses. Preliminary analyses were carried out to ensure a sufficient amount of sequencing data and the absence of outliers ascribable to unknown factors. In summary, three biological replicates were kept for all samples, with the exception of D0 and D48 (VO), were four samples were available, and PL-D94 where only two samples could be included.

The comparisons among groups were performed with the empirical analysis for differential gene expression tool, based on the “Exact Test” developed by Robinson and Smyth^32^ and incorporated in the EdgeR package^33^. First, the biological replicates of VO and PL samples were compared to FO at D48 and D94, to evaluate the differential effects of the three feeds on PC and LV expression profiles. Moreover, to assess the shared effect of dietary supplementation in the two tissues, all samples (VO + FO + PL) collected at D48 were treated as replicates and compared to D0. A similar comparison was carried out between D94 and D48. Differentially expressed genes were identified based on a false discovery rate (FDR)-corrected p-value (according to the Benjamini–Hochberg procedure) threshold of 0.05, combined with a fold change value higher than + 2 (for up-regulated genes) or lower than −2 (for down-regulated genes).

Histograms, Principal Component Analysis (PCA), scatter plots and heat maps were built using gene expression levels represented as transcripts per million (TPM), as suggested by Wagner *et al*. (2012) to ensure efficient data normalization and comparability both within and between samples. Heat maps were constructed using a hierarchical clustering of features approach based on Euclidean distance and average linkage criteria. Log_10_-transformed TPM values were used for all plots^34^.

### Validation of expression levels by qRT-PCR

Eight immune-related genes identified as differentially expressed in pyloric caeca upon first feeding (i.e. in the transition between D0 and D48) were selected for validation by qRT-PCR experiment. The genes selected were those encoding a C1q-like protein (CIGSSA_093400), the interferon inducible protein 44 – IFI44 (CIGSSA_017505), immunoglobulin Mu - IgM (CIGSSA_017459) and Tau - IgT (CIGSSA_093863) heavy chains, a macrophage-inducible C-type lectin (CIGSSA_114990), the bactericidal/permeability-increasing protein - BPI (CIGSSA_109332), the nucleotide-binding oligomerization domain-containing protein 1 – NOD1 (CIGSSA_115242) and a class I histocompatibility antigen-like protein (CIGSSA_122127). As an internal control for normalization we used elongation factor 1 alpha, which is regarded as a stable housekeeping gene in several teleosts and is validated as a suitable reference gene for qRT-PCR in Atlantic salmon^35,36^. The list of primers used is shown in Table 2. Whenever possible, the primers were designed to obtain an amplicon size optimal for RT-PCR (80–120 bp) and to minimize self-complementarity.

**Table 2:** Primers used for validation by qRT-PCR.

Sequence Name	Sequence 5′ to 3′	Accession no.
EF1A_F	CTGTCGGTGTCATCAAGGCT	BT043567.1
EF1A_R	GCTCAGGTTTTGAGATGCCG	
C1q-like_F	CAGTTACAGGGGCCTTCACA	CIGSSA_093400
C1q-like_R	ATGCGGTTGTCCATACCAGT	
IFL44_F	ACCCCCGTCAACAAGACTTC	CIGSSA_017505
IFL44_R	CCACCCCAAGGTGTTTTCCT	
IgMu heavy chain_F	ATCTGAAGCTGTCTGGTCTGAG	CIGSSA_017459
IgMu heavy chain_R	GCGCAAGAGGGAACAAAGTC	
macrophage-inducible C-type lectin_F	TCATCCCACAAAGAGACCCAG	CIGSSA_032594
macrophage-inducible C-type lectin_R	GTGTTCTCTCCACAAGCCCA	
PGRP-S_F	ACTGTCGGCTGCAACTGTTA	CIGSSA_032594
PGRP-S_R	GTCGTCCATATGCTGGGAGG	
NOD1_F	TGACACACGACATGCTACGG	CIGSSA_115242_1
NOD1_R	TCCACAGTCCACCGAGTCTA	
IGT_F	GGGACACAAGTCACCGTCTC	CIGSSA_093863
IGT_R	TGGACAAAGTCTGTCAGCGG	
Class I histocompatibility antigen-like protein_F	TGGACACACAGACTCGTTC	CIGSSA_122127
Class I histocompatibility antigen-like protein_R	CAGGTTTTTGCTTTGGCTGAC	
BPI_F	GGGACCGTTTCAGGTTGGAT	CIGSSA_109332
BPI_R	TTGCATGTCTCGTTGGTTTGAT	

One µg of total RNA extracted from the salmon individuals subject to RNA-sequencing was used as a template for cDNA synthesis with a QuantiTect Reverse Transcription Kit (Qiagen, Hilden, Germany) according to manufacturer’s instructions. cDNAs from both pyloric caeca and liver were diluted 10 times and used as templates for RT-PCR assays. The qRT-PCR reactions were carried out on a LightCycler® 96 instrument using LightCycler 480 SYBRgreen I Master kit (Roche Applied Science, Germany). Each sample was run with three technical replicates. Following an initial denaturation step at 95 °C (1 minute), the PCR reaction program included 40 amplification cycles, as follows: 10 s denaturation at 95 °C, 10 s annealing at 55 °C and 10 s elongation at 72°c. Efficiency of PCR reactions were calculated with the LinRegPCR gene quantification software (Heart Failure Research Center, Netherlands) and mean Ct values were used to calculate statistically significant gene expression changes across groups with the Qbase + software (Biogazelle Co., Belgium).

### Pathway analysis

The gene ontology (GO) annotations associated with the Atlantic salmon genome were downloaded from Salmobase and imported in the CLC Genomics Workbench. To investigate the biological effects of the diets, we performed gene set enrichment analyses, based on a hypergeometric test on annotations, on the subsets of differentially expressed genes (DEGs) at each time point. The thresholds for detection of significant alteration of GO biological processes, molecular functions and cellular components were set at 1 × 10^−5^ (p-value) and 3 (observed – expected), respectively.

**Figure 1:**
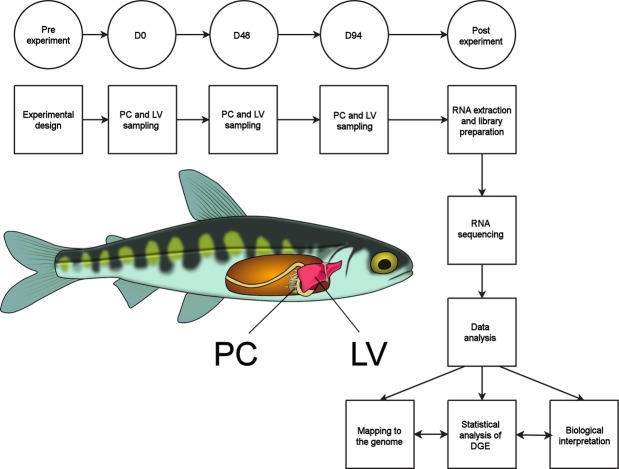
Schematic diagram depicting the workflow of the study. The pyloric caeca (PC) and liver (LV) of an Atlantic salmon individual in the fry stage are shown.

To gather further insights into the modulation of biological pathways related to immunity and inflammation, the list of immunity-related differentially expressed genes detected at D48 in the pyloric caeca (Supplementary Table 1) was subjected to an Ingenuity Pathway (IPA, Qiagen, Hilden, Germany) core analysis. First, gene annotations were extracted based on the Uniprot ID of the top BLAST hit listed in the Salmobase annotation file. Whenever possible, Uniprot IDs were converted to human Entrez gene IDs to allow the import of gene expression data to the IPA environment. Due to the widespread presence of paralogous genes in the salmon genome^24^, whenever redundant annotations were detected, only the feature displaying the lowest FDR-corrected p-value was kept for subsequent analyses. The outputs of the core pathway analysis were used to generate network maps for significantly modulated pathways, which were manually edited to reflect sequence divergence between salmon and human. The schematic workflow of the study is illustrated in Fig. 1.

### Ethical statement

This fish experiment has been approved by the Norwegian Food Safety Authority (Mattilsynet), and by the Ethics Committee at Norwegian University of Science and Technology (Trondheim, Norway). Fish transfer, breeding, maintenance and sampling procedures have been carried out in accordance with the regulations contained in section 11 (“regulations on the use of animals in experiments”) of the Lovdata legal resources repository. All the procedures for water supply and water quality, oxygen and nitrogen compounds, pH and salinity, temperature, light and noise, fish density and environmental complexity, feeding and handling were carried out in accordance with regulations for fish experiments provided by Mattilsynet. In order to follow the regulations on animal welfare, we considered all requirements for anesthetic and pain management, permitted killing methods in accordance with regulations specified in section 16, paragraph 2 (Lovdata). To improve the quality of life of the animals subjected to experimental trials, we have done our best to prevent stress and suffering during handling and sacrifice.

## Results and Discussion

### Sequencing output

The raw sequencing output obtained was in line with expectations, with individual libraries ranging from 13 to 26 million reads, averaging 18.5 million reads (Supplementary Table 2). A similar sequencing depth has been previously demonstrated to be appropriate for RNA-seq-based gene expression studies associated with nutrition in salmon^37^. The trimming procedure, applied to remove sequencing adapters, low quality nucleotides and residual reads shorter than 90 nucleotides led, on average, to the removal of 0.46% of the reads per library. The mapping step resulted in the successful alignment of 62.7 and 63.6% clean reads for pyloric caeca and liver, respectively, to the annotated regions of the salmon reference genome. The relatively large fraction of unmapped reads, in line with results obtained from previous studies carried out on the same species^29^, was most likely linked to the presence of non-annotated features in the salmon genome, as well as to sequence polymorphisms between the reference genome and the salmon individuals subject of the present study.

### The switch from exogenous to endogenous feeding leads to a relevant immune regulation in the pyloric caeca, but not in the liver

Our whole-transcriptome approach potentially allows the study of the effects of different feeding regimes on salmon fry on various biological levels. These range from the biochemical regulation of digestive processes, to the influence of nutrients on fish growth, development and metabolic performance. These effects have obvious implications for salmon farming. The present part of the study focus on the modulation of immune pathways and inflammatory processes in response to composition of the diet (i.e. FO, VO and PL feed) over the first 94 days after the complete internalization of the yolk sac. Indeed, understanding whether any feed composition has immunomodulatory properties is an aspect of primary importance for the management of salmon hatcheries during the developmental phases with transition from juvenile to adult life.

In this study, the choice of the two tissues was made to select two organs that, in spite of the massive impact on metabolic pathways related to nutrition and, more in general, on fish physiology, were also expected to display largely different behaviors in terms of immunological response, both functionally and temporally. The fish digestive system is the key point of contact with pathogens and, as such, it is associated with GALT (gut-associated lymphoid tissue), and plays a fundamental role in mucosal immunity^38^. The pyloric caeca is therefore expected to be highly responsive to nutritional changes, as well as to undergo important remodeling along fish development, in particular in correspondence with the switch from endogenous to exogenous diet and the first contact with foodborne microbes. On the other hand, while the liver is not recognized among the main fish immune tissues, it can be used to infer immune information due to its richness in peripheral blood that contains mature leukocytes, as suggested by different studies^39,40^. Although this tissue is involved in the maintenance of immune homeostasis, unlike gut, primarily based on adaptive antigen recognition, presentation and T/B lymphocyte activation processes, it is primarily linked with specific aspects of the innate immune system, such as the production of acute phase proteins^41^.

**Figure 2:**
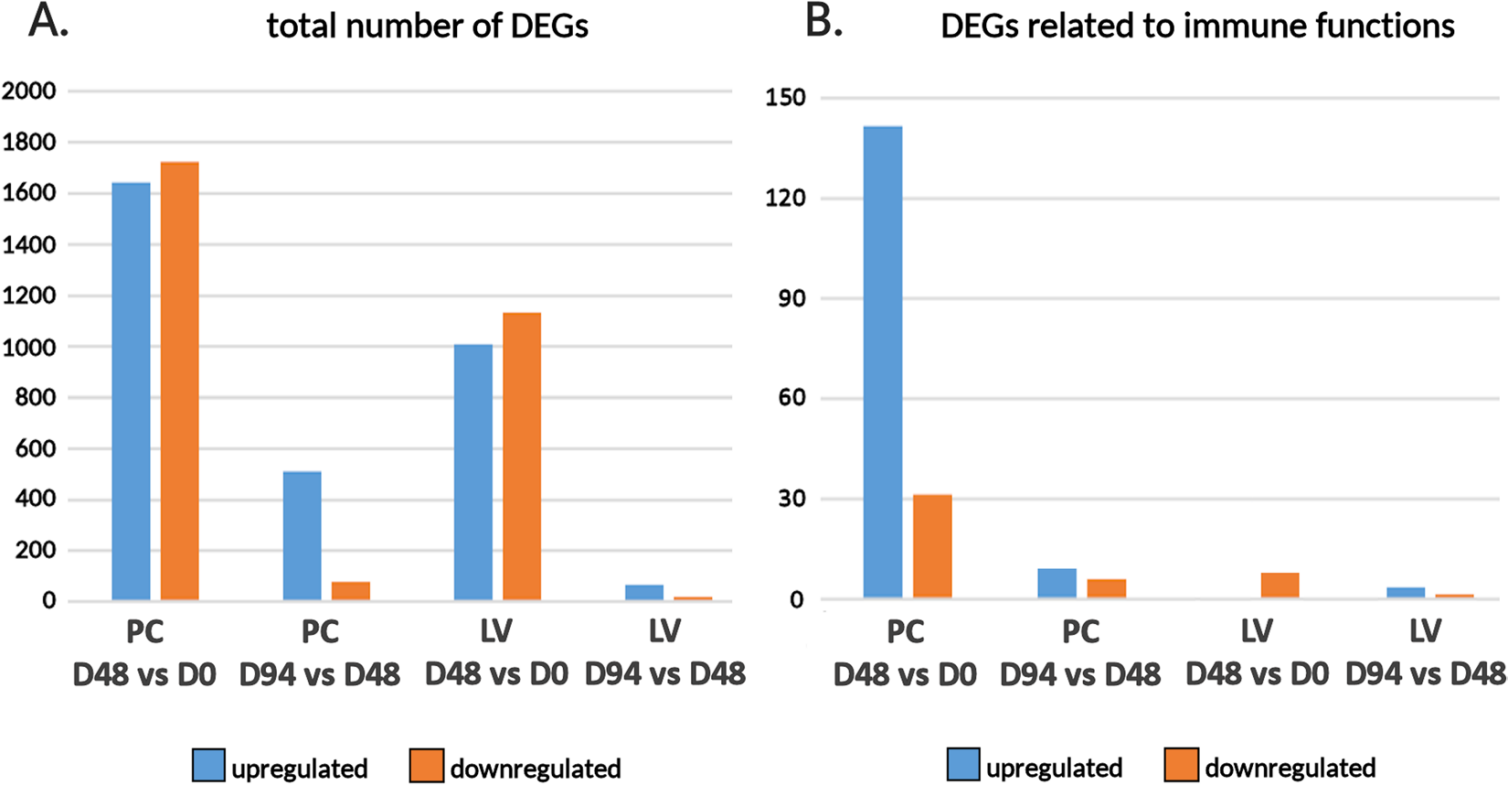
Panel A: general overview on the number of DEGs identified by the statistical analysis of differential expression in the comparison between time points (D48 vs D0 and D94 vs D48), in pyloric caeca and liver tissues, regardless to the type of feeding used. Panel B: subset of DEGs whose annotation was linked to immune functions (Supplementary Table 1). Scatter plots showing pairwise comparisons between D48 and D0, and between D94 and D48, in both tissues are detailed in Supplementary Fig. 3. The complete list of immune DEGs is available in Supplementary Table 1.

In this experiment, many genes were identified as transcriptionally modulated in a time-dependent manner in both tissues, independent of the type of diet (Fig. 2, panel A). The largest number of DEGs was observed in PC at D48, where the number of up- and down-regulated genes peaked at 1,643 and 1,725, respectively. A much lower number of genes were further modulated at the following experimental time point (D94), where 512 transcripts were up-regulated and 77 downregulated. Although gene regulation in the liver was less impacted by fist feeding than the PC, more than 2,000 genes showed important modulation during the first 48 days (1,009 up-regulated and 1,134 down-regulated, respectively) (Fig. 2, panel B). Like in PC, the extension of the feeding up to D94 only revealed minor modifications in the expression profiles, as just 65 and 20 additional genes were subject to up- and down-regulation.

The inspection of the annotations prominently associated with the DEGs identified in the aforementioned comparisons revealed the modulation of a broad range of biological processes, molecular functions and cellular components. In PC, the most enriched of the 60 Gene Ontology terms over-represented after 48 days (Supplementary Table 3A) were linked to mRNA transcription and translation processes (including several structural components of the ribosome). This may be due to the massive tissue remodeling occurring in this organ in the transition from the alevin to the fry stage, which is expected to require a massive energetic investment in protein synthesis. These annotations were followed, as expected, by functions intimately related to the switch from endogenous to exogenous nutrition, including lipid metabolic processes (e.g. lipid and cholesterol transport, and triacylglyceride metabolism), response to nutrients and digestion. Interestingly, several annotations related to immune processes (which will be discussed in detail in the following sections), were also strongly enriched in the DEG set. A detailed investigation of DEG annotations revealed that more than 170 genes involved in immune processes were significantly modulated at the 48 h time point, evidencing a relevant impact of first feeding on the functionality of the immune system in salmon PC.

Coherent with the lower number of DEGs identified in the transition between D48 and D94, only 7 associated enriched annotations could be identified (Supplementary Table 3B). This suggest that all major transcriptional alterations took place within the first 48 days from the switch from endogenous to exogenous diet.

The liver was also sensibly impacted by first feeding, as pointed out by the 69 annotations significantly enriched associated to the 2,143 DEGs identified at D48 (Supplementary Table 3C). The GO terms over-represented in LV after first feeding were in large part linked with fatty acid metabolism and biosynthesis, oxidation-reduction processes and mitochondrial energetic processes, in line with the primary role this tissue covers in the storage of fatty acids and secretion of lipoproteins. Compared to PC, no annotation linked to the immune system was detected. The analysis of the few DEGs modulated at the subsequent time point (D94), could only identify three significantly enriched GO terms (Supplementary Table 3D).

In light of the data from LV, it was clear that this tissue, in spite of the dramatic alteration of processes related to growth and metabolism, did not undergo significant immunomodulation in response to first feeding. Consequently, while the in depth analysis of the modulation of gene expression in response to first feeding in the liver might be of great interest for nutritional studies, this specific aspect will not be assessed in the present study, which will be focused on the highly immune-responsive PC tissue.

### The dietary supplementation of FO, VO or PL has little effect on the expression profile of immune genes in the pyloric caeca

As the PC was identified as the most attractive target tissue for the investigation of the immune-modulatory properties of first feeding, an important question to address was whether FO, VO and PL diets had a differential effect on the modulation of genes involved in immune and inflammatory processes.

We investigated the effect of feed composition at the two time points (D48 and D94) and found a moderate impact in terms of number of differential expressed genes, with 71 DEGs detected at both time points. On both days the largest differences were observed between VO and PL diets, followed by the FO and VO diets. Only minor changes were observed in the comparison between FO and PL (Supplementary Fig. 1). No statistically significant enrichment of annotations related to immune response and inflammation was found in the analysis of these DEGs.

Most importantly, feed composition displayed a marginal effect on the modulation of genes impacted by first feeding (outlined in the section above), as 99.1% and 98.1% of these were not affected by the composition of the diet during the first 94 days after the alevin-to-fry transition (Supplementary Fig. 1). More in detail, only one out of the previously described immune genes modulated by first feeding at D48 was differentially expressed in response to diet. Specifically, CIGSSA_029629, a macrophage-inducible C-type lectin was up-regulated 2.4 times in PL-fed salmon compared to VO-fed salmon. No immune system related genes were affected by the type of diet in the comparison between transcriptional profiles at D48 and D94.

These results were unexpected, as the most important compositional difference among the three feeds was linked to the relative content of long chain n-3 fatty acids (EPA and DHA) in FO and PL, as opposed to the presence of ALA and LA in VO diet. It was expected that this difference would influence the modulation of epithelial and systemic immune response^42^.

Only a few studies have previously investigated the effects of VO and FO dietary components on fish immune performance, reporting conflicting results in different tissues and different farmed fish species. For example, some authors observed a significant decrease in circulating leukocytes in VO-fed salmon, together with other hematological alterations possibly connected to a detrimental effect on immune system functionality^43,44^. However, those studies did not investigate the effects on mucosal immunity in the digestive tract, as they were limited to blood and liver. In another study, Montero and colleagues^45^ reported that substitution of FO with VO enhanced expression of pro-inflammatory cytokines and impaired the immune defense in response to bacterial infections in the gilthead seabream intestine and head kidney.

Data collected in other species suggest that the type and amount of n-3 and n-6 fatty acids in feeds, rather than their source, have an important role in the determination of the physiological outcome. For instance, dietary intake of ALA/LA feed with a 0.5:1 ratio can beneficially affect fish innate immune function and growth. Whether the presence of monounsaturated FAs, saturated FAs and n-6 fatty acids from vegetable sources can alter the endogenous metabolism of long chain n-3 fatty acids, resulting in a change of the profile of prostaglandins and inflammation response remains an open question.

Our experimental results showed that the type of fatty acid supplemented with the diet had a negligible effect on the modulation of salmon immune genes in PC in the first 94 days after the alevin-to-fry transition. However, the long-term consequences are presently not known.

### A 48-day feeding period determined a marked increase in the expression of immune-related genes in the pyloric caeca

**Figure 3:**
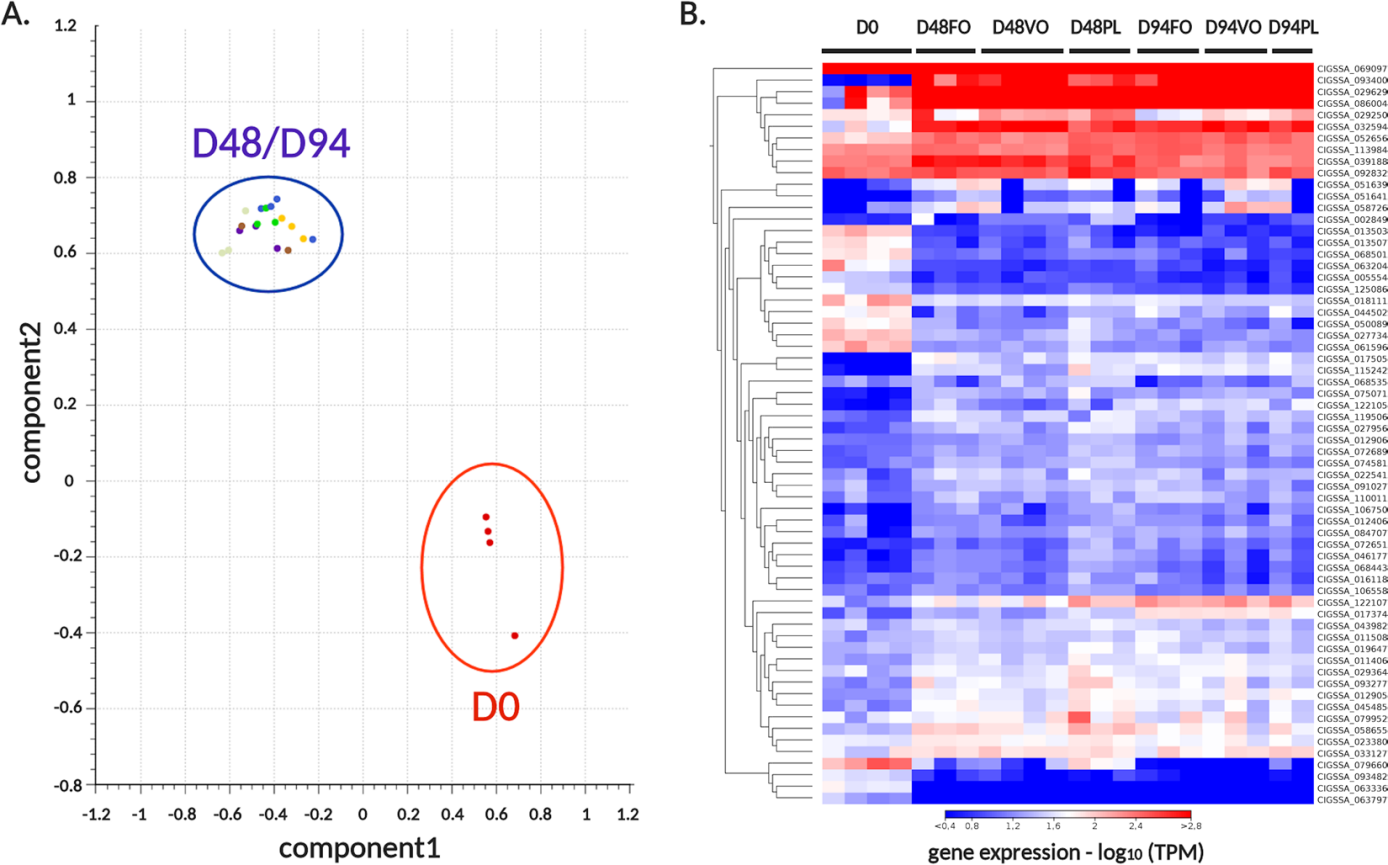
Panel A: PCA of gene expression profiles of the immunity related gene set in PC. Log_10_-transformed TPM expression values were used to generate the PCA plot. Panel B: hierarchical clustering of a subset of differentially expressed immune genes in PC, based on Log_10_-transformed TPM expression levels. Clustering was performed using a Euclidean distance and average linkage. Gene IDs are indicated at the right-hand side of the figure and the corresponding expression levels are provided in Supplementary Table 1.

As mentioned above, we observed a significant change in regulation of 175 genes involved in immune system function in PC at D48 (Fig. 2, panel B, Supplementary Table 1) across all three diets. The overwhelming majority of these (144, corresponding to 82%) were up-regulated. The immune modulation occurring at this time point was also supported by the significant over-representation of gene ontology, such as “immune response” (p-value = 9.17 E^−7^), “cellular response to cytokine stimulus” (p-value = 4.4E^−7^), “immune effector process” (p-value = 4.4 E^−5^), “cytokine production involved in immune response” (p-value = 1.15 E^−4^), inflammatory response (5.57 E^−4^) and others. Only 12 genes related to immune system response were further significantly modulated in PC from D48 to D94 (7 upregulated, 5 downregulated). Due to the massive involvement of immune-related genes in the transcriptional response of PC to first feeding, possibly explained by the high responsiveness of the mucosal immune response of fish intestinal epithelium^46,47^, this tissue was subjected to in-depth analyses to dissect the most relevant molecular players involved. The detailed inspection of immune gene expression trends confirmed the remarkable similarity between D48 and D94, whereas D0 emerged as a highly divergent sample (Fig. 3). The poorly significant alterations detected in the comparison between D48 and D94 and the overlapping signature of expression of immune genes (Fig. 3), point out that a major reprogramming in the expression of immune genes might have occurred in the early phases of the feeding program, i.e. before D48. D0 was set at the transition between the alevin and fry developmental stages, consistent with the switch from endogenous nutrition from the yolk sac to external diet. While previous studies have revealed that salmon alevins possess a well-developed and potentially functional digestive system one week before the full absorption of the yolk sac in the abdominal cavity^23^, the switch to exogenous nutrient sources undoubtedly represents a key point in the maturation of the mucosal immune system of the intestine. Although the fish intestine presents an associated microbiota since the very early stages of development, due to the contact with surrounding water and yolk sac digestion^48^, the first external feeding induces profound changes in the magnitude and composition of this microbial community. Moreover, this switch exposes the digestive tract for the first time to exogenous food particles, stimulating the expression of several immune genes, as previously reported for rainbow trout^49^. Other studies evidenced that salmon intestine only undergoes minor histomorphological changes during the fry stage, in agreement with the finding that the extension of the feeding to 94 days did not lead to significant additional alterations of the expression profiles of immune genes in our experiment (Fig. 3).

### A broad range of immune cells and processes in the pyloric caeca are activated by first feeding

**Figure 4:**
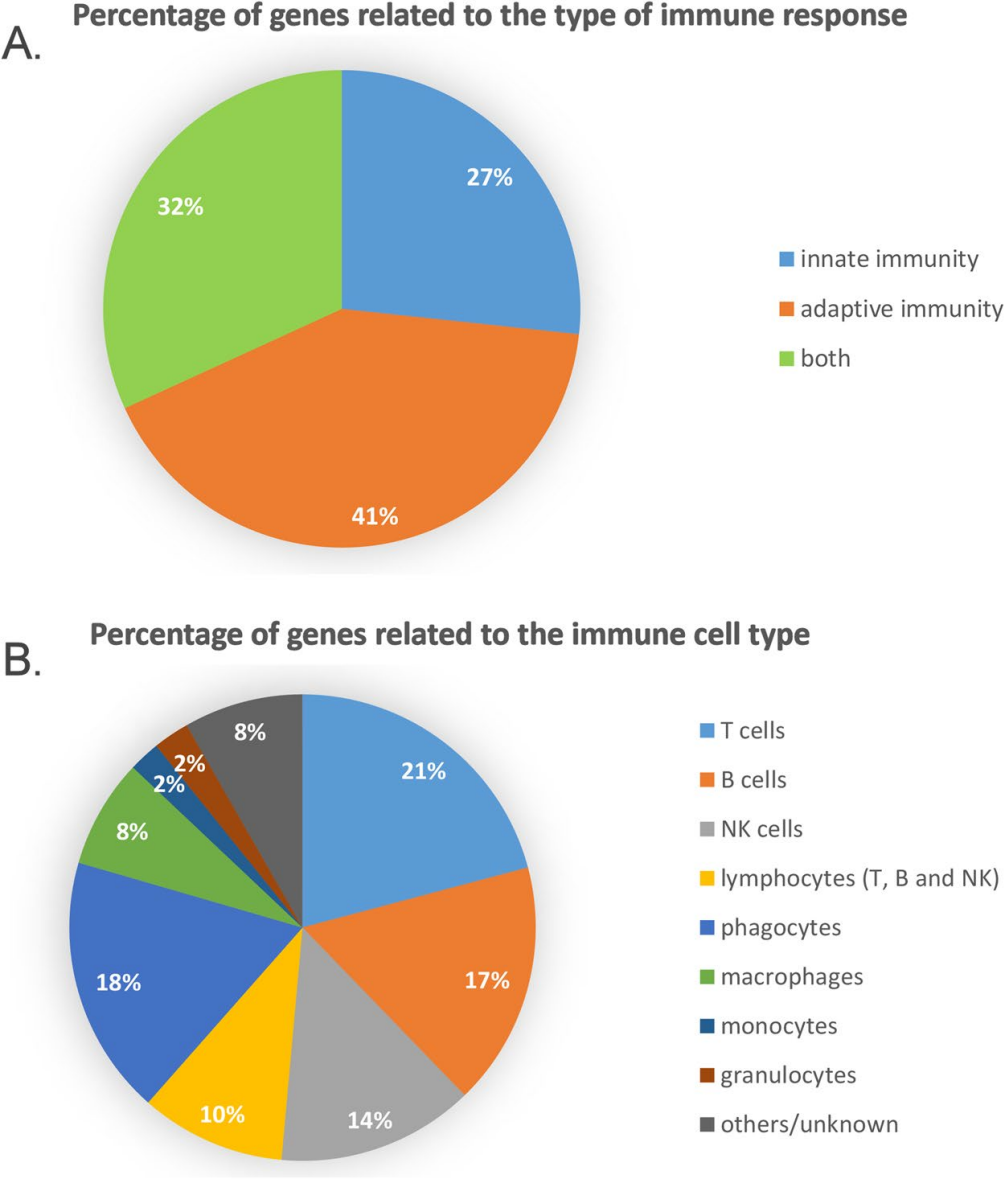
Panel A: Contribution of innate and adaptive immune response to the upregulated DEGs evidenced in pyloric caeca at D48 (see Fig. 2B). Panel B: Involvement of different immune cell types in the modulation of gene expression in pyloric caeca at D48. “Phagocytes” indicates genes whose function could not be unequivocally connected to any of phagocyte subpopulation.

We investigated whether the marked signatures of the immune response outlined above for PC at D48 might be connected to the involvement of any particular immune cell type and whether it could be explained by innate or adaptive processes. Both “non-specific” pattern recognition receptors (PRRs) and antimicrobial effectors shared by all metazoans, and typical components of the vertebrate adaptive immune response (e.g. immunoglobulins) responded with comparable trends to the switch from endogenous to exogenous nutrition (Fig. 4, panel A). A clear overlap was detected between the innate and adaptive vertebrate immune system, as 56 genes involved in each of the systems were significantly upregulated. As discussed in detail in the following sections, this observation is consistent with the massive activation of the fundamental pathways such as the complement system, opsonization and phagocytosis, which act as functional bridges between innate and adaptive immunity, enabling an integrated organismal response to challenges^50^.

No dominant transcriptional signature of any specific immune cell type could be identified, although the mRNAs displaying the most significant shifts could be connected to the activity of either lymphocytes or phagocytes (Fig. 4, panel B). Within the former category, a slight prevalence of T cell-related transcripts over those linked to B and NK cell functions was observed. Among the genes more intimately linked to specialized phagocytic cell populations, those connected to macrophages were clearly prevailing over monocytes and neutrophils.

Overall, these observations highlight that the highly significant upregulation of immune genes observed in PC upon first feeding is not simply explainable as a transitory acute phase response to the feeding regime switch, but rather as a persistent potentiation of the immune system, as it was maintained with minor variations up to D94.

### A mechanistic hypothesis about the modulation of the immune system in pyloric caeca upon first feeding with lipid-rich diets

The predictive causal network-based analysis we performed with IPA can be useful to formulate mechanistic hypotheses^51^, which might in part explain the transcriptional reprogramming of immune genes that we observed in PC at D48. These considerations are particularly valid for highly conserved biological pathways whose mechanisms of regulation are shared by all vertebrates, as the Ingenuity Knowledge Base only contains information on human, mice and rat models. IPA identified the complement system as the most significantly regulated canonical pathway within the first 48 days of feeding (p-value = 5.52E^−12^, activated based on a z-score = 0.45), followed by PRR-mediated microbial recognition (p-value = 8.12E^−10^, strongly activated based on a z-score = 2.33). TREM-1 signaling (p-value = 2.09E^−9^, also strongly up-regulated based on a z-score = 2.83) and Th1 and Th2 activation pathway (p-value = 1.49E^−8^, no direction). Interestingly, also the interleukin-10 signaling pathway and the activation of interferon regulatory factor (IRF) from cytosolic pattern recognition receptors were predicted as significantly regulated pathways (Table 3).

**Table 3:** Ingenuity Pathway Analysis summary.

Name	p-value
**Top canonical pathways**	
Complement System	5.52 E^−12^
Role of Pattern Recognition Receptors in Recognition of Bacteria and Viruses	8.12 E^−10^
TREM-1 Signaling	2.09 E^−09^
Th1 and Th2 Activation Pathway	1.49 E^−08^
Neuroinflammation Signaling Pathway	2.20 E^−08^
**Top Upstream Regulators**	
LPS - activation	1.64 E^−34^
TNF - activation	1.64 E^−19^
poly rI:rC-RNA - activation	2.91 E^−19^
IFNG - activation	5.59 E^−19^
TLR4 - activation	2.74 E^−17^

The interpretation of these results is not straightforward due to a limited functional conservation between salmon and human genes, and the presence of paralogous genes in salmon originating from whole genome duplication. Some general insights can, however, be drawn from the indications provided by IPA. The transcriptional modulation observed is consistent with a strong activation of the immune system by microbe associated molecular patterns (MAMPs) such as lipopolysaccharide (LPS) and poly (I:C). These were indeed predicted as the top upstream regulators candidates (with p-values = 1.64E^−34^ and 2–9E^−19^, respectively), with the consequent dimerization of Toll-like receptors (TLRs) leading to the recruitment of their cytosolic adaptors MyD88 (p-value = 3.69E^−15^) and TICAM1 (p-value = 2.08E^−13^), followed by the activation of important transcriptional regulators such as NF-κβ (p-value = 7.65E^−13^) and IRF3 (p-value = 3–59E^−13^).

The migration of these transcription factors to the nucleus would in turn increase the production of pro-inflammatory cytokines, such as IL-1 beta, IL-2, IL-4, IL-6, IL-10, CSF-2, interferon alpha (p-value = 4.45 E^−17^) and gamma (p-value = 5.59 E^−19^), and the activation of their receptors, leading to the recruitment of different immune cell types. At a cellular level, the transcriptional profile observed at D48 in PC is consistent with a highly significant activation, migration and adhesion of circulating immune cells, leukocytes in particular (p-value = 1.22 E^−39^), with an activation of inflammatory response (p-value = 2.47 E^−31^). The involvement of the TREM-1 signaling is also strongly consistent with the activation of the inflammatory response, through the action of pro-inflammatory transcription factors such as STAT3/5 and NF-κβ.

### The complement system as a key player in the potentiation of immune response

**Figure 5:**
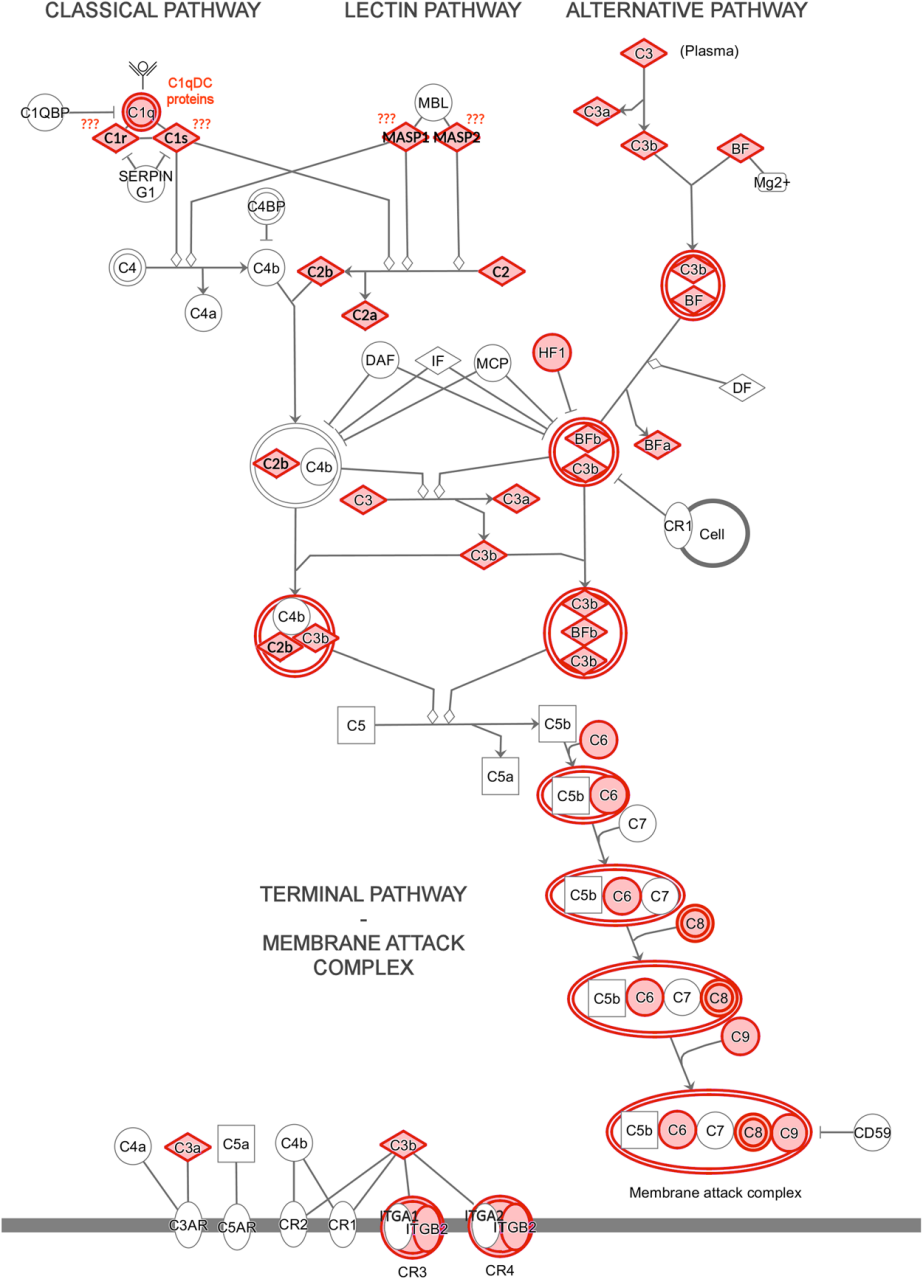
Schematic organization of the three branches of the complement system, i.e. the classical pathway, the lectin pathway and the alternative pathway with reference to the human complement system. Genes identified as upregulated in the pyloric caeca at D48, based on the criteria specified in the materials and methods section, are highlighted in red. Components of the pathways whose orthology status with human counterparts is presently uncertain/unknown are indicated by red question marks.

Within this complex picture, it remains to be established how the dietary switch is triggering this strong potentiation of immune and inflammatory response in juvenile salmons. We show that the complement system, a major player acting at the crossroads between innate and adaptive immunity, emerges as a key candidate for explaining a large fraction of the observed modifications, due to its highly significant activation at D48 (IPA canonical pathway analysis p-value = 5.52 E^−12^). Like in all metazoans, the fish complement system plays a pivotal role in the innate humoral defense response to infection by the sensing of opsonized pathogens or damaged cells^52^. As exemplified in Fig. 5, a large fraction of its molecular components was, to some extent, up-regulated in PC, while they were unaltered in LV during the same time frame. Three different pathways can potentially trigger the complement system in teleost fish: the classical pathway (CCP), the alternative pathway (ACP) and the lectin pathway (LCP). The CCP involves the interaction of antigen-complexed immunoglobulins with the C1q complex; the LCP implies the intervention of mannose binding lectin (MBL) or ficolins in pathogen-associated carbohydrate recognition, without the mediation of antibodies; the ACP is triggered by the spontaneous hydrolysis of the component C3 in response to pathogen recognition^53^.

Although the C2/factor B (gene ID: CIGSSA_058617), the C3 components (Gene ID: CIGSSA_029250) and the regulator factor H (gene ID: CIGSSA_05216) showed a remarkable up-regulation, they are not sufficient to unequivocally support the activation of the ACP. This is due to the stable expression of factor D, a key regulating serine protease. Moreover, little evidence supports the activation of LCP. Indeed, while a MASP-like (mannose-associated serine protease, gene ID: CIGSSA_029636) transcript, expected to cleave C2 in response to MAMP recognition by MBL or ficolins, was significantly up-regulated (Fig. 5), it was not possible to conclude whether it was orthologous to MASP-1/MASP2, or to C1r/C1s. These latter two serine proteases share a common evolutionary origin with MASPs, but they are involved in the cleavage of complement component C4 in CCP. Furthermore, no convincing homolog to MBL, ficolins or other lectins possessing an N-terminal collagen region could be found among the DEGs at D48.

**Figure 6:**
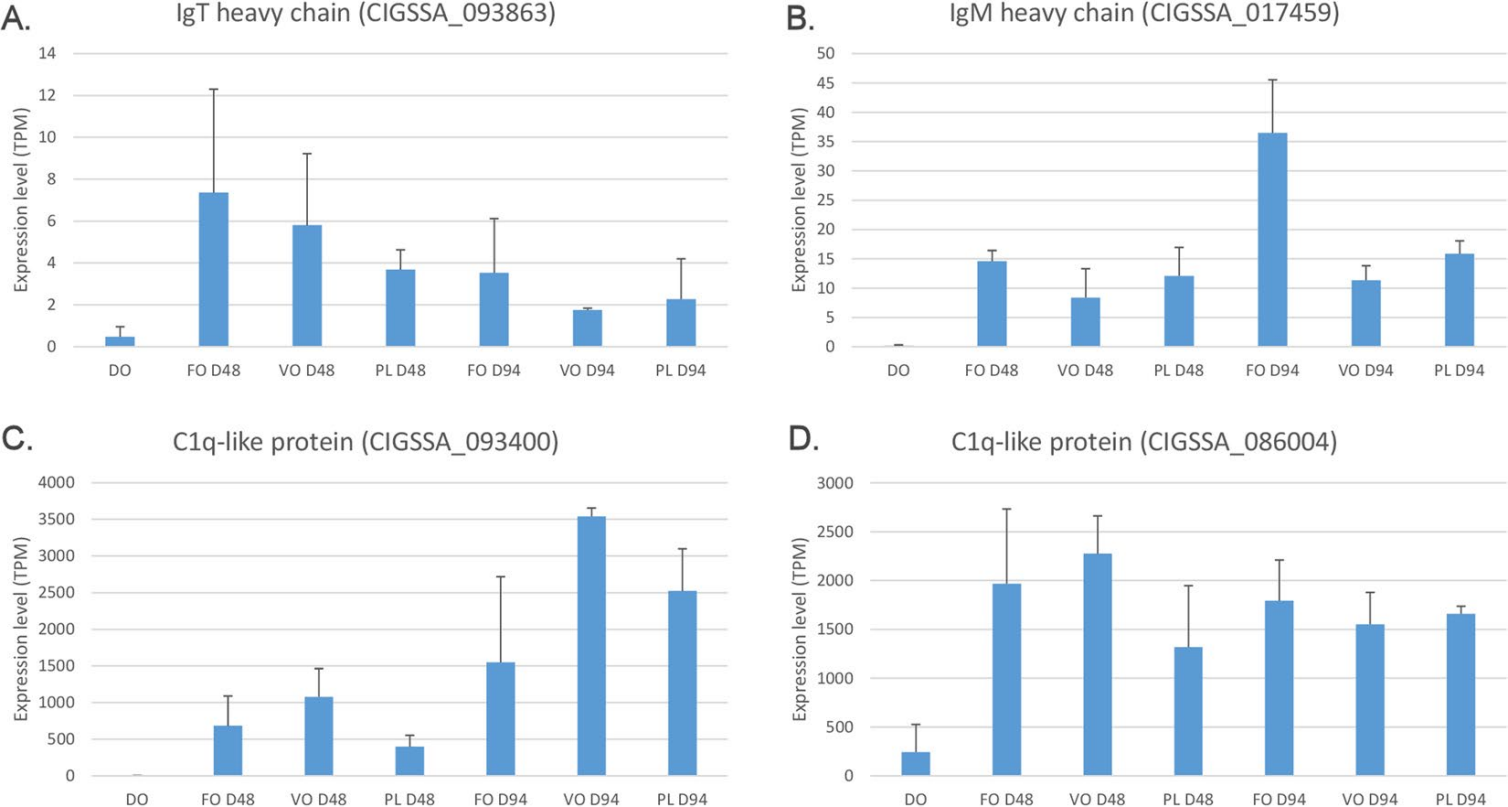
Gene expression trends of immunoglobulin Tau heavy chain (Panel A), immunoglobulin Mu heavy chain (Panel B) and two C1q-like proteins (Panels C and D) genes during the time course experiments. Histogram bars represent *in silico* calculated gene expression values (indicated as Transcripts Per Million), plus the standard deviation of the available biological replicates. None of the four genes represented here were detected as differentially expressed in the comparison among the three feeding regimes (see Supplementary Fig. 1).

Based on our observations, the most likely route of activation of the complement system in our experiment might reside in the CCP (Fig. 5). This hypothesis is supported by the 10-fold up-regulation of a gene sharing significant homology to the three main chains of the C1q complex (gene ID: CIGSSA_058852). The impressive shift in the expression of other C1q-like genes, such as CIGSSA_093400, showing a 380-fold increase, CIGSSA_090078 (62-fold increase) and CIGSSA_086004 (13-fold increase), was even more suggestive (Fig. 6), and was further confirmed by a qRT-PCR approach (Supplementary Fig. 2). Although these C1q-domain containing (C1qDC) proteins only show little sequence homology with the three chains of the human C1q complex, the marked immune recognition properties of this highly plastic recognition domain have been well documented in animals^54,55^. Moreover, some multifunctional C1qDC proteins, such as adiponenctin, can modulate the activity of the classical pathway in human and we cannot exclude a similar role for the salmon C1qDC genes^56^.

The involvement of CCP was supported also by the high number of significantly up-regulated genes encoding immunoglobulin (Ig) light and heavy chains. Although the complex organization of Ig light chain loci^57^ currently prevents a detailed assessment of the genes specifically involved in this response, we can confirm that both immunoglobulin Mu (gene ID: CIGSSA_017459) and immunoglobulin Tau (gene ID: CIGSSA_093863) heavy chain genes were strongly up-regulated, by 50 and 14 times, respectively at D48 (Fig. 6). This result was further confirmed by qRT-PCR analysis (Supplementary Fig. 2). IgT is thought to be an immunoglobulin of the utmost importance in mucosal immunity, with a key role in the opsonization of intestinal bacteria^58^, but its relationship with the CCP is still poorly understood. On the other hand, the functional link between IgM and the activation of the complement system has been well-established in teleost by previous studies. In fact, IgM represents the most abundant immunoglobulin type in fish serum, where it plays a role in both innate and adaptive immune response. Specifically, the effector functions of IgM include complement activation and the subsequent opsonization and phagocytosis of invading pathogens^59,60^. Previous studies have revealed that IgM reached high concentrations in the serum of adult salmonids (1 to 10 mg/mL serum, depending on the health status and rearing condition^61^) and that its production is developmentally regulated, as this Ig type is only detectable at low levels (<0.1 mg/mL) at early larval development^62^. Our study is not the first one to show an enhancement of IgM production in fish in response to dietary supplementation, as a similar response has been demonstrated in the channel catfish due to amino acid addition^63^.

Finally, it is important to remark that the activation of the complement system also involves some components of the terminal pathway, i.e. the membrane attack complex (MAC), devoted to microbial killing through lytic mechanisms. In detail, we could detect up-regulation of C6, C8 and in particular C9, the main structural component of the pore ring. The concerted stimulation of MAC and other effector molecules that are part of the innate immune system might provide a highly efficient system of protection against microbial invasion in juvenile salmons subjected to lipid-rich diets.

### An overview on relevant markers of the modulation of the intestinal immune system in response to exogenous nutrition

As outlined in the previous sections, the modulation of the immune and inflammatory responses in salmon fry attained in our experimental trial is the product of a complex network of cellular and molecular interactions, and involves a large array of components (Supplementary Table 1). Consequently, several of the genes involved only show marginal fluctuations, which barely meet the threshold criteria selected for detecting differential expression, while others, possibly playing a major role in immune response, show much stronger alterations, both in terms of fold change and FDR-corrected p-value.

**Figure 7:**
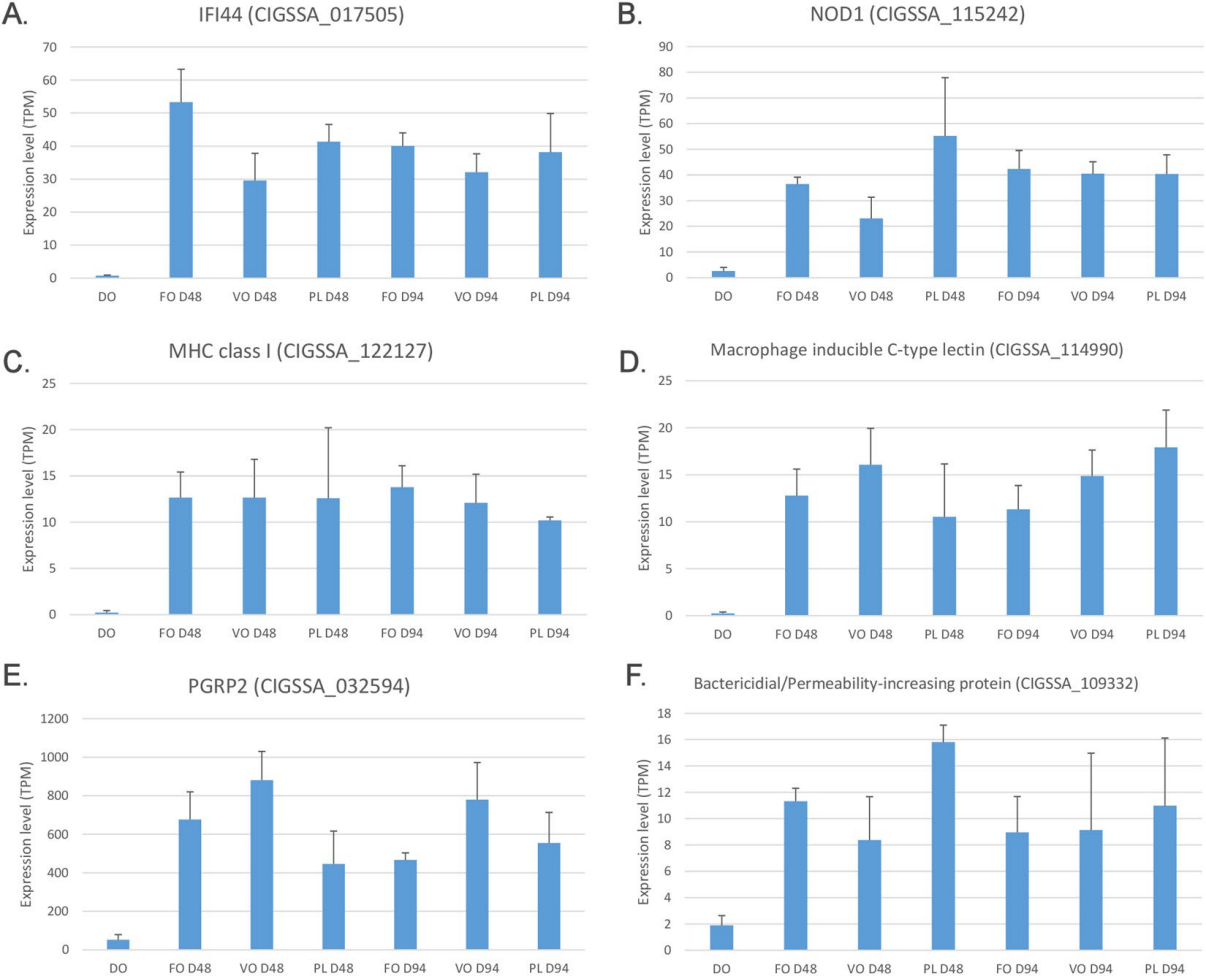
Gene expression trends of interferon-inducible protein 44 (Panel A), nucleotide-binding oligomerization domain-containing protein 1 (Panel B), MHC class I protein antigen (Panel C), macrophage-inducible protein (Panel D), peptidoglycan-recognition protein 2 (Panel E) and bactericidial/permeability increasing protein (Pane F) genes during the time course experiments. Histogram bars represent *in silico* calculated gene expression values (indicated as Transcripts Per Million), plus the standard deviation of the available biological replicates. None of the six genes represented here were detected as differentially expressed in the comparison among the three feeding regimes (see Supplementary Fig. 1).

Below a few such examples are briefly discussed due to their high relevance and potential implications in the potentiation of the immune response. The first case is IFI44, identified by the statistical analysis as one of the most significantly up-regulated genes in PC at D48 (FDR-corrected p-value = 6.9 E^−33^, FC = 66.05, Fig. 7, panel A), and confirmed as a significant DEG by qRT-PCR (see the next section for details). IFI44 is an interferon alpha-inducible protein, whose expression is associated with the infection by several viruses and whose role is linked with the suppression of viral transcription^64^. The marked overexpression of IFI44, together with the modulation of other cytosolic viral sensors (e.g. RLR1), might provide an increased competence to respond to viral infections in juvenile salmons.

NOD1 represents a second key example of upregulation (FDR-corrected p-value = 1.41 E^−18^, FC = 19.68, Fig. 7, panel B) confirmed by qRT-PCR (see the next section for details). This intracellular PRR acts as a sensor of bacterial peptidoglycans (PGN), but can also be involved in the detection of protozoans and viruses. It stimulates the expression of NF-κB, determining the activation of an inflammatory response and the production of antimicrobial effectors^65^. The fundamental and conserved role of NOD1 has been confirmed in teleost^66^, suggesting that its upregulation in salmon might effectively provide an enhanced ability to recognize and eliminate invading microbes in the gut.

A third example is CIGSSA_122127, a gene encoding the alpha chain of a MHC class I protein. This gene was strongly upregulated (FDR-corrected p-value = 1.62E^−12^, FC = 50.14, Fig. 7, panel C), together with several other genes encoding other members of the MCH class I superfamily. Like in other vertebrates, the function of teleost class I MHC is to display fragments of digested non-self peptides to cytotoxic T cells through the endogenous pathway^67^. Furthermore, some lineages of class I MHC genes play an important role in the antigen cross-presentation pathway in fish leading to the activation of CD8 + T-cells, in particular in species where MHC class II genes have been lost^68,69^.

Fourth, we also detected several upregulated genes homologous to the macrophage inducible C-type lectin (Mincle), with CIGSSA_114990 as the most significant in terms of fold change (FDR-corrected p-value = 2.19 E^−12^, FC = 51.29, Fig. 7, panel D). Mincle is considered to be a multifunctional player in immunity, due to its ability to recognize pathogens and damaged cells and to induce the production of cytokines^70,71^

Finally, some of the genes with the most significant increases in gene expression were antimicrobial effectors of the innate immune system. One example is the peptidoglycan recognition protein 2 (PGRP2) homolog, that reached expression levels close to 1,000 TPM (FDR-corrected p-value = 5.84 E^−18^, FC = 21.26, Fig. 7, panel E). This gene product, characterized by an amidase enzymatic activity towards PGN, is induced by bacterial challenges and might therefore be involved in bacterial sensing and killing^72^. This protective function has been clearly demonstrated in teleost^73,74^, and in particular against Gram-positive bacteria, whose cell wall is richer in PGN.

Another relevant example is the BPI protein, that was similarly strongly over-expressed (FDR-corrected p-value = 1.84 E^−10^, FC = 8.52, Fig. 7, panel F), as validated by qRT-PCR (see the next section for details). This antimicrobial effector selectively binds to LPS, determining the specificity of recognition for Gram-negative bacteria, and leads to bacterial cell lysis^75^. The conserved antimicrobial function of fish BPIs, demonstrated by multiple studies^76–78^, implies that its induction in salmon might provide increased protection against Gram-negative bacterial infection. This activity could be complementary to that of lysozyme, expected to confer increased protection against Gram-positive bacterial infections.

### Validation of RNA-seq results with qRT-PCR

We selected eight genes involved in immune functions significantly up-regulated in PC during the first 48 days of exogenous feeding for validating the gene expression alterations identified with our transcriptomic approach. Six of the genes analyzed displayed expression trends consistent with those evidenced by RNA-seq, i.e. they were significantly up-regulated at D48 compared to D0, only undergoing minor fluctuations at D94 (Supplementary Fig. 2). The similarity between the outcomes of the two methodologies was particularly striking for the immunoglobulin Mu (CIGSSA_017459) and Tau (CIGSSA_093863) heavy chains, and for the C1q-like protein (CIGSSA_093400). These genes experienced massive upregulation (Fig. 6 and Supplementary Fig. 2), as they were expressed as barely detectable levels at D0 (IgM was not even detectable by qRT-PCR at D0). The marked upregulation of the bactericidal/permeability-increasing protein (CIGSSA_109332), the interferon inducible protein 44 (CIGSSA_017505) and the nucleotide-binding oligomerization domain-containing protein 1 (CIGSSA_115242), showing slightly higher basal expression levels at D0, was similarly confirmed with high confidence (Fig. 7 and Supplementary Fig. 2).

Although in some cases the calculated expression and fold change values varied between the two methodologies, the discrepancies observed were in line with the known systemic differences existing between the two quantification methods^79^. Factors such as the different dynamic ranges of the two methodologies and inter-individual sequence variability might explain the unsuccessful amplification of another target gene (the macrophage-inducible C-type lectin, CIGSSA_114990) in some samples. Similarly, allelic variation might have impaired an efficient primer design for a class I histocompatibility antigen-like protein (CIGSSA_122127), leading to discordant results between RNA-seq (where a strong upregulation was detected) and qRT-PCR (where no significant differences were evidenced).

The results obtained from LV (data not shown) confirmed the very poor (and often undetectable) expression of immune genes in this tissue, reinforcing the indications pointing towards a lack of modulation during the time course of the experiment.

## Conclusions

Overall, the marked enhancement in the expression of genes involved in the recognition and elimination of viral, bacterial and eukaryotic pathogens indicate a broad activation of the immune system in the digestive tract of salmon fry in the shift from endogenous to exogenous diet. Due to the involvement of immunoglobulins, these changes might provide a long-term potentiation of immune defense against infection. Preliminary indications collected from this pilot study suggest that these effects are independent from feed composition, as negligible differences were observed between VO, FO and PL-rich diets.

In-depth studies should be planned to explore in detail the dynamics of immune gene expression during the earlier stages of development, to investigate the responsiveness of the mucosal immune system in salmon within the first few days after the exposure to the new dietary regime. It is also important to note that no significant alteration of the immune-related transcriptome could be detected in liver, although massive alterations were observed for other biological pathways. This suggests that the potentiation of the immune response specifically occurs within the digestive system and cannot therefore be considered as a generalized effect at the whole-organism level. This is natural because most interaction with microbes is expected to take place at the mucosal surfaces for healthy individuals. While previous studies have reported the positive regulation of a few immune molecular markers by qPCR is salmonids within a similar time frame^49^, this is, to the best of our knowledge, the first study to provide a global overview of the regulation of the immune transcriptome following first feeding in the Atlantic salmon.

We can exclude the occurrence of viral or bacterial infections in the tanks used for the experiment, as the physiological variables of salmons were monitored during the entire experiment and no histological alteration could be detected upon dissection. Therefore, the most likely hypothesis to explain the observed gene expression changes has to be found in the first contact of the digestive tract with food particles and, consequently, with massive amounts of non-self antigens of food and bacterial origin. The alevin-to-fry transition is indeed an important phase in the development of the immune system in salmon that like other teleost, largely depends on innate mechanism^80,81^ and maternal immunity^82,83^ in earlier larval stages, due to the delayed development of lymphomyeloid organs.

Our study suggests that first feeding leads to a fast and remarkable stimulation of the innate and adaptive immune response in salmon fry, coinciding with the absorption of the yolk sac and the depletion of maternally-derived immune molecules, through a process which involves the CCP as a key biological pathway.

## Supplementary information

Supplementary Figures

Supplementary Table 1

Supplementary Table 2

Supplementary Table 3

## Data Availability

Raw sequencing data has been deposited in the NCBI SRA database and is available upon request to the authors.
