# Supplementary material for: Dietary fatty acid source has little effect on the development of the immune system in the pyloric caeca of Atlantic salmon fry
Source: Sci Rep. 2019 Jan 10;9:27. doi: 10.1038/s41598-018-37266-3 (PMC6328623; doi:10.1038/s41598-018-37266-3)
Supplement: Supplementary file 1 — Supplementary Figures [file 41598_2018_37266_MOESM1_ESM.docx]

**Dietary fatty acid source has little effect on the development of the immune system in the pyloric caeca of Atlantic salmon fry**

Mahsa Jalili, Yang Jin, Atle M. Bones, Yngvar Olsen, Olav Vadstein, Mari-Ann Østensen, Francesco Buonocore, Marco Gerdol, Alberto Pallavicini, Giuseppe Scapigliati

**Supplementary Figure 1**


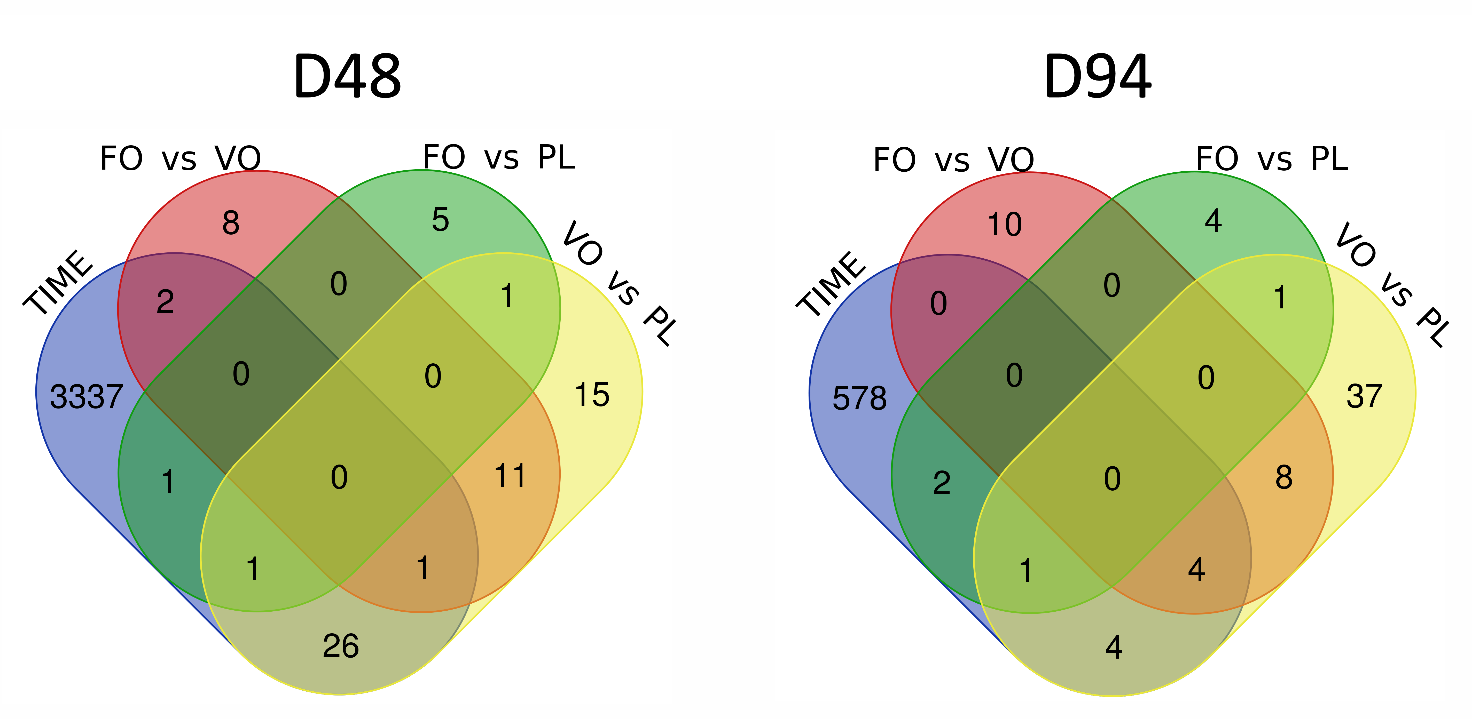


Venn diagrams displaying the number of differentially expressed genes (DEGs) identified by statistical analyses in the pyloric caeca at D48 (left panel) and D94 (right panel). FO, VO and Pl identify the fish oil, vegetable oil and phospholipid-rich diets, respectively. “TIME” indicates the number of DEGs modulated in the comparison among time points (i.e., between D48 and D0 in the left panel, D94 vs D48 in the right panel).

**Supplementary Figure 2**


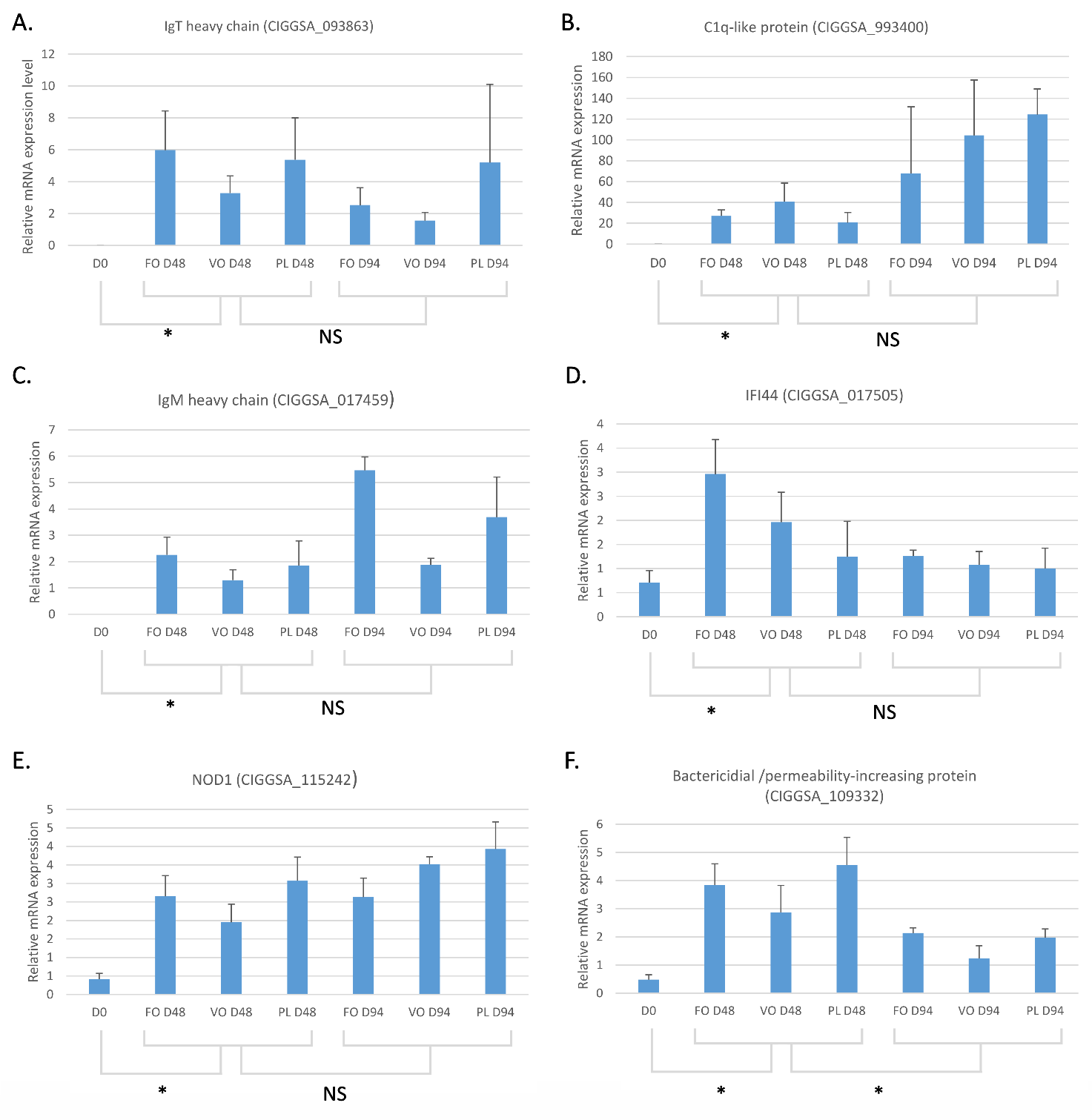


Validation of the overexpression of six selected immune-related genes upon the switch from endogenous to exogenous diet by qRT-PCR. Selected genes are immunoglobulin Tau heavy chain (panel A), C1q-like protein (panel B), immunoglobulin Mu heavy chain (panel C), IFI44 (panel D), NOD1 (panel E) and bactericidial/permeability-increasing protein (panel F). Relative expression values were calculated based on the expression value of the stable housekeeping gene elongation factor 1 alpha.

Statistically significant differences between D48 and D0, and between D94 and D48 are indicated by an asterisk. NS: not significant. Each bar represents the mean + SEM obtained from two to four biological replicates per sample (see the materials and methods section for details). The expression level for each biological replicate was calculated based on three independent technical replicates.

**Supplementary Figure 3**


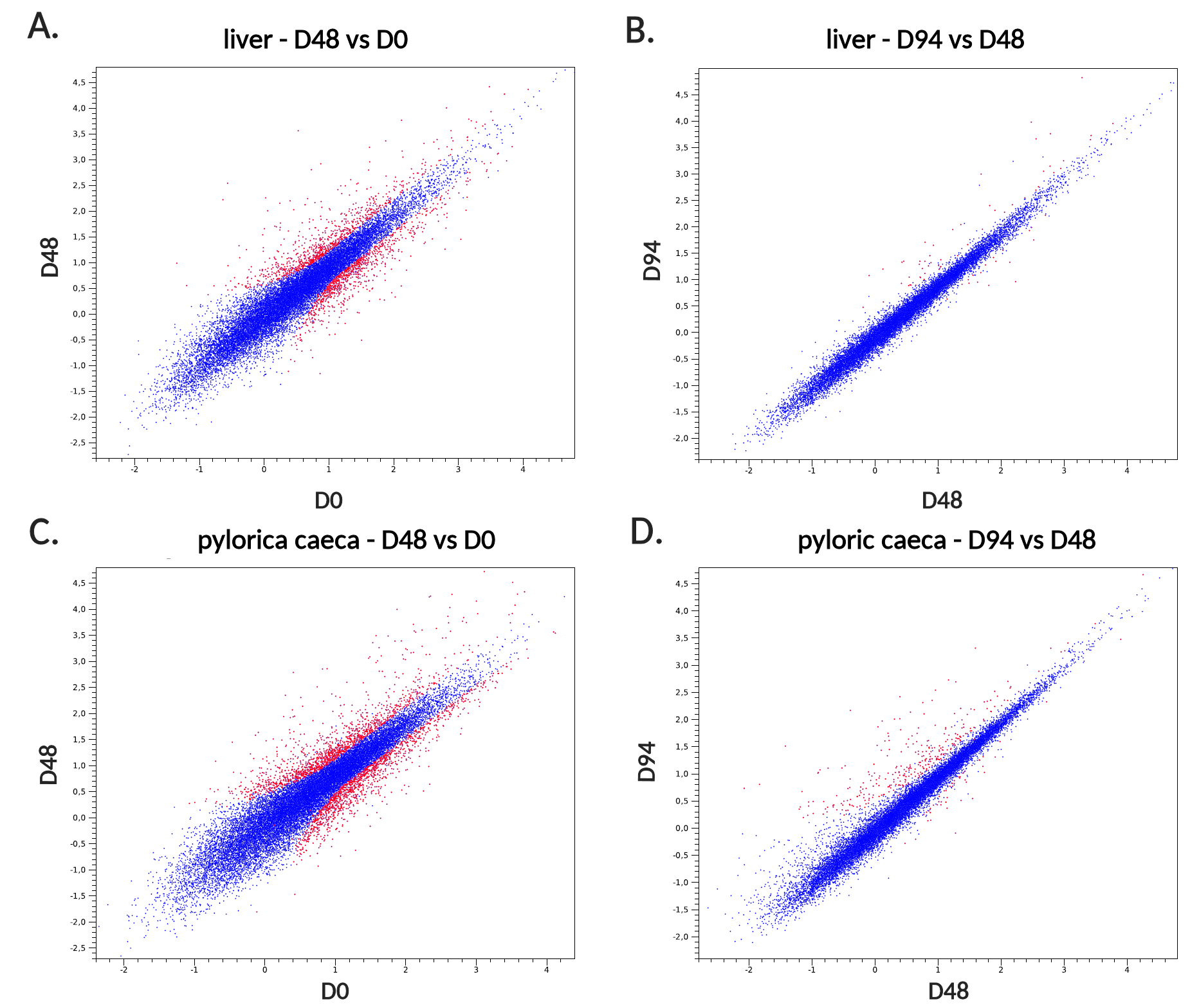


Scatter plots displaying a comparison between gene expression profiles of liver and pyloric caeca (D48 vs D0 and D94 vs D48). Differentially expressed genes are shown in red. Plotted expression values are log_10_ –transformed TPM.
